# Effect of network size on comparing different stock networks

**DOI:** 10.1371/journal.pone.0288733

**Published:** 2023-12-14

**Authors:** Kamrul Hasan Tuhin, Ashadun Nobi, Md. Jafar Sadique, Mahmudul Islam Rakib, Jae Woo Lee

**Affiliations:** 1 Department of Computer Science and Telecommunication Engineering, Noakhali Science and Technology University, Noakhali, Bangladesh; 2 Department of Physics, Inha University, Incheon, Republic of Korea; Roma Tre University: Universita degli Studi Roma Tre, ITALY

## Abstract

We analyzed complex networks generated by the threshold method in the Korean and Indian stock markets during the non-crisis period of 2004 and the crisis period of 2008, while varying the size of the system. To create the stock network, we randomly selected N stock indices from the market and constructed the network based on cross-correlation among the time series of stock prices. We computed the average shortest path length L and average clustering coefficient C for several ensembles of generated stock networks and found that both metrics are influenced by network size. Since L and C are affected by network size N, a direct comparison of graph measures between stock networks with different numbers of nodes could lead to erroneous conclusions. However, we observed that the dependency of network measures on N is significantly reduced when comparing larger networks with normalized shortest path lengths. Additionally, we discovered that the effect of network size on network measures during the crisis period is almost negligible compared to the non-crisis periods.

## 1. Introduction

The complexity of the stock market has been a subject of extensive research in recent decades [1–5]. Graph theory has proven to be a valuable tool for gaining insights into the structural and functional characteristics of stock market networks. However, when comparing the topologies of different networks using graph theory, several significant limitations arise.

One such limitation is that stock market network measures are dependent on the number of nodes, which means that different network sizes may exhibit varying measure values. Furthermore, experimental network topologies are often unknown, which can further complicate direct comparisons of network properties. When comparing stock networks of different sizes, the bias caused by network size differences can result in incorrect conclusions.

Wu et al employed graph theory and the vector autoregressive method to study the stock markets of ASEAN5, China, Japan, and South Korea [6]. Their findings indicated that despite government efforts to promote financial market coordination and integration in East and Southeast Asia, these markets were not as robust as they appeared. Nobi et al generated threshold networks, hierarchical networks, and minimum spanning trees from a correlation matrix constructed using 185 individual stock prices [7]. They demonstrated that during crisis periods, the degree distribution of the largest cluster in threshold networks was thicker than during non-crisis times. In the Korean and US stock markets, non-financial companies served as central nodes of the minimal spanning tree during the crisis period, while financial firms occupied the center nodes during non-crisis periods [8]. Nobi et al also investigated changes in correlation and network structure between 2000 and 2012 using 145 stock prices from the Korean stock market and 30 indices from international stocks [9]. Their findings revealed that average correlations of international indices increased over time, while average correlations of local indices decreased, except for significant fluctuations during crises when they surpassed average correlations of global indices.

Several studies have employed graph theory and neural networks to predict stock market prices, demonstrating superior performance to traditional approaches by incorporating structural information into the prediction models [10, 11]. Dimitrios et al investigated the linkages among companies on the Greek Stock Exchange from 2007 to 2012 and found that the market was "shallow," meaning that a small number of powerful investors had a significant impact on the volatility of numerous company values [12]. Wen et al constructed a tail dependence network using stock market data from 73 countries, revealing that European markets were more influential than Asian and African markets during both booms and recessions, and that geographically neighboring economies were susceptible to financial risks [13]. Hu et al analyzed Shanghai and Shenzhen A-share stocks using threshold networks, and they determined that Chinese stock networks exhibited the properties of a small-world network and followed a power-law distribution [14]. Van Wijk et al employed graph theory to compare brain networks of varying sizes and connectivity densities [15]. Their study aimed to identify network measures that are not influenced by changes in network size and connectivity density. Although there are currently no effective methods to account for the size and connectivity density-dependent impacts, the authors found that certain measures, such as the normalized path length and non-normalized clustering coefficient, are less sensitive to changes in network size and connectivity density.

Some articles have conducted comparative analyses of stock markets of varying sizes [16–18]. Eom et al constructed minimum spanning trees (MSTs) for 463 stock indices from KOSPI and 400 stocks from S&P 500, and then compared the network properties of both MSTs [16]. They concluded that network degree is the most valuable quantity because it can describe network topology and has a close relationship with the market index. Alam et al investigated the stock correlations and their index effects for developed, developing, and emerging markets, using 377 stocks from S&P 500, 165 stocks from KOSPI, and 220 stocks from the Dhaka Stock Exchange [17]. They found that during the global financial crisis, stocks in developed and emerging markets were more strongly correlated than those in developing markets. Balash et al used 194 stocks from IMOEX, 163 stocks from CSI163, and 468 stocks from S&P 500 to conduct network analyses of Russian, Chinese, and US stock markets [18]. They compared the structural and topological properties of these networks and reported that the topological properties of Russian stocks differ from those of Chinese and US stocks. However, in all of these studies [8, 9, 14, 16–18], the bias induced by the size difference of the network was overlooked, as most network properties are subject to change with network size. Hence, comparing network properties of differently sized networks directly can lead to inconsistent outcomes.

In this study, we investigate how network measures are affected by network size and propose various methods to overcome the inconsistency associated with comparing stock market networks of different sizes. We also examine how network properties respond to changes in network size during both crisis and non-crisis periods. Specifically, we analyze the network properties of 500 companies on the Korean stock market and 500 companies on the Indian stock market and observe how these properties change with variable network size and market stability. To reduce the dependence of network properties on changing network size, we apply normalization techniques. Our results indicate that normalizing with the range of possible values significantly reduces the dependence of the average shortest path length on network size, while variable network size has little influence on the average clustering coefficient. Therefore, normalized average shortest path length and non-normalized average clustering coefficient can be used to compare stock networks of different sizes with minimal bias. We also find that during crises, the impact of network size on network metrics is relatively low compared to non-crisis periods, which can be helpful in identifying critical market timelines. Furthermore, we discover that the network properties of larger networks are not greatly influenced by their size. The primary objective of this research is to investigate various strategies for correcting network measures for their dependence on number of nodes to enable comparison of stock networks of different sizes with minimal bias, which has not been explored previously.

The remaining sections of the paper are structured as follows: Section 2 discusses the data sources used in this study to construct networks. In section 3, we outline the methods used to generate threshold networks from stock correlations, define various network measures, and describe normalization techniques. We then present and discuss our findings in section 4. Finally, we summarize our study and draw conclusions in section 5.

## 2. Data description

We consider stock market data from two different countries to conduct our study: the Korean Composite Stock Price Indexes (KOSPI) and the National Stock Exchange of India Limited (NSE). The data was collected from Yahoo Finance and Investing(https:// https://finance.yahoo.com/world-indices) [19, 20]. The KOSPI is a set of indexes that reflect the overall Korean Stock Exchange and its constituents. On the other hand, the NSE is the largest financial market in India and a reflection of the Indian Stock Exchange as a whole. We chose 500 indices from KOSPI and 500 indices from NSE during the years 2004 and 2008, with 2008 being the year of the global financial crisis. The purpose behind selecting these years is to examine how well our assessment holds up during times of financial crisis as well as times when there is no crisis.

## 3. Network construction and normalization methods

We calculate the log returns of daily closing prices by first taking the natural logarithm of the daily closing prices and then subtracting the result of the previous day’s closing price from the result of the current day’s closing price. Returns essentially represent the profit or loss resulting from an investment within a specific timeframe, and they measure the amount of money gained or lost as a result of investing. If we denote the daily closing price of stock *i* at time *t* as *p*_*i*_(*t*) and daily returns of stock *i* at time *t* as *r*_*i*_(*t*), then the log returns *r*_*i*_(*t*) over period *Δt* can be written as,

$$r_{i}(t)=ln[p_{i}(t)]-ln[p_{i}(t-\Delta t)]$$ (1)

where *i* = 1,…,*N* and *t* = 1,…,*T* where *N* is the total number of stocks and *T* is the number of days. Since we are only considering daily returns, we set *Δt* to one day. We use a one-year time window to divide the daily returns before constructing the network. Next, we calculate the cross-correlation matrix for all the logarithmic returns *r*_*i*_(*t*) within a one-year segment using the following formula,

$$c_{ij}=\frac{\left\langle r_{i}(t)*r_{j}(t)\rangle -\langle r_{i}(t)\rangle *\langle r_{j}(t)\right\rangle }{\sigma_{i}*\sigma_{j}}$$ (2)

where *c*_*ij*_ is an element of the cross-correlation matrix and <∙> represents the mean and *σ*_*i*_ represents the standard deviation of the stock *i*.

After the correlation matrix is formed, a stock network is typically created using a fixed threshold technique, which is a commonly used method in stock market research [9, 21–23]. However, there are other approaches to constructing stock networks. For instance, some studies have employed Minimum Spanning Trees (MST) using correlation matrices [24–27], while others have used Planar Maximally Filtered Graphs (PMFG) [28, 29]. In a limited number of cases, researchers have utilized machine learning techniques to discover network relationships and then applied thresholds to construct networks [30].

Each element in the correlation matrix corresponds to an edge in the network. For example, the edge *e*_*ij*_ connecting nodes *i* and *j* corresponds to the correlation matrix element *c*_*ij*_. The fixed threshold technique utilizes a threshold correlation value *θ*, where |θ|≤1, to determine which edges to include in the final network. If the absolute correlation value between two stocks exceeds the threshold, for example, if |*c*_*ij*_|>*θ*, an edge is added between those stocks, and the corresponding entry in the adjacency matrix is set to one. We set a threshold value of 0.3 for constructing our networks, which was chosen without any specific reasoning or prior knowledge to ensure that it did not influence our analysis. We also tested other threshold values to determine their impact on our results, but the findings remained stable across all tested threshold values, and we did not observe any significant variation in our outcomes (not shown in the paper). Despite this, we used a threshold value of 0.3 to generate our results, as it offered a suitable balance between capturing the network’s structure while keeping it relatively simple. To eliminate self-connections, we set all diagonal elements of the adjacency matrix to zero.

Various network measures that provide insights into the network topology can be determined. We used the small-world index to identify the small-world structure of Korean and Indian stock networks. A small-world network is characterized by the property that the average shortest path lengths between nodes increase slowly as the network size grows. The small-world index can be utilized to determine the small-world property of a network. Small-world networks are distinguished by their path lengths and clustering coefficients [31]. The clustering coefficient indicates the level of interconnectivity among nodes in the immediate neighborhood. A clustering coefficient of *C* = 1 indicates a fully connected neighborhood, whereas a value close to *C* = 0 indicates a sparse neighborhood. The average clustering coefficient of a network of size *N* is calculated using the following formula [32],

$$C=\frac{1}{N}\sum_{i=1}^{N}\frac{\sum_{1\leq j\leq l\leq N}a_{ij}a_{jl}a_{il}}{k_{i}(k_{i}-1)/2}$$ (3)

where *k*_*i*_ denotes the degree of node *i*, and *a*_*ij*_ is the element of the network’s adjacency matrix.

The average shortest path length is the average number of steps along the shortest pathways between all possible pairs of nodes in a network. It is defined as [33],

$$L=\frac{1}{n(n-1)}\sum_{i\neq j}d(V_{i},V_{j})$$ (4)

where *d*(*V*_*i*_, *V*_*j*_) denotes the shortest path length between nodes *V*_*i*_ *and V*_*j*_.

To reduce the sensitivity of network properties to the size of the network (*N*), different normalization techniques are employed. In some studies, graph measurements are normalized by comparing them to those of random networks with the same number of nodes and edges [34, 35]. In this approach, the average path length (*L*) and the average clustering coefficient (*C*) of the stock network are normalized by dividing them by the corresponding values of a random network. We used Erdos-Renyi random networks for normalization of stock network measures. The normalization process can be expressed mathematically as follows,

$$\tilde{L}=\frac{L}{L_{rand}}\;\mathrm{and}\;\tilde{C}=\frac{C}{C_{rand}}.$$ (5)

The stock network exhibits properties that lie between those of lattice and random networks, and thus possesses small-world properties. Consequently, the path length and clustering coefficient of a stock network will fall between those of a lattice and a random network [36]. Therefore, these measures can be normalized by considering the range of possible values between lattice and random networks using the following equation.


$$L^{\prime }=\frac{L-L_{rand}}{{L_{lattice}-L}_{rand}}\;\mathrm{and}\;C^{\prime }=\frac{C-C_{rand}}{C_{lattice}-C_{rand}}.$$ (6)

In this context, the lattice network is created using a regular ring lattice, while the random network is formed using an Erdos-Renyi random graph. After normalization, the small-world index can be defined using the clustering coefficient and the path length as follows,

$$\sigma =\frac{\tilde{C}}{\tilde{L}}.$$ (7)

If the value of the small-world index is greater than 1, then the network is considered a small-world network [31, 37]. We find different values of *σ* from various sized Korean stock networks, and on average, the value of *σ* = 14.95 is obtained during the non-crisis period of 2004, and *σ* = 1.55 is obtained during the crisis period of 2008. In the case of Indian stocks, on average, the value of *σ* = 2.07 is obtained during the non-crisis period of 2004, and *σ* = 1.21 is obtained during the crisis period of 2008. This means that during the non-crisis period, both Korean and Indian stock networks have a much more small-world structure, while during the crisis period, the networks have a loose small-world structure because the small-world index is lower during the crisis.

The small-world index indicates whether a network exhibits a small-world structure, with values greater than *σ* = 1 indicating such a structure [31, 37]. We computed the values of *σ* for Korean and Indian stock networks of varying sizes, and on average, during the non-crisis period of 2004, the Korean stock networks had a high small-world index with *σ* = 14.95, while during the crisis period of 2008, the networks showed a weaker small-world structure with *σ* = 1.55. Similarly, during the non-crisis period of 2004, the Indian stock networks exhibited a small-world structure with *σ* = 2.07, and during the crisis period of 2008, the networks displayed a less pronounced small-world structure with *σ* = 1.21. This suggests that during the non-crisis period, both Korean and Indian stock networks had a more pronounced small-world structure, while during the crisis period, the networks exhibited a less distinct small-world structure, as reflected by the lower values of the small-world index.

## 4. Results and discussions

### 4.1 Network structure

**Fig 1:**
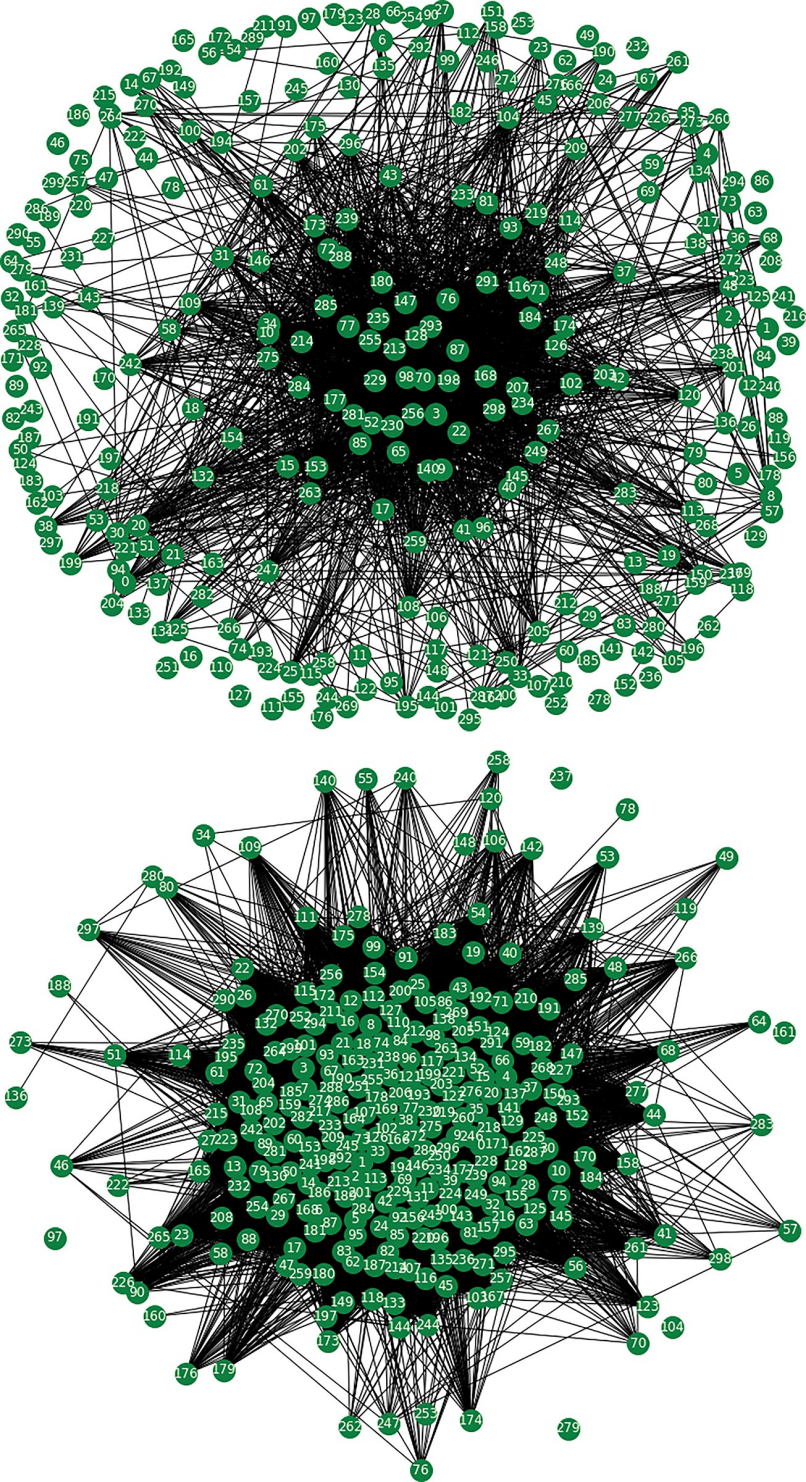
The network structure of the Korean stock market with 300 nodes (stocks), including (a) the year 2004, a non-crisis period, with 1,781 edges, and (b) the year 2008, during the crisis, with 25,848 edges. A significant difference in the compactness of the network is observed between the crisis and non-crisis periods. To generate the stock network, we randomly selected 300 stocks and applied the threshold method.

The complex network presented in this study is generated using the correlation matrix of returns from the Korean and Indian stock markets. The threshold method is applied to obtain the network structure. Fig 1 illustrates two stock networks, where each node represents a stock, and an edge connecting two nodes signifies a significant correlation between them. In Fig 1(A), the network is generated using the threshold method on 300 randomly selected stock indices from the Korean stock market in 2004. The resulting network contains a giant cluster with *N*_*_gaint*_ = 183, as well as some small, isolated clusters. However, the network is sparse, and many detached nodes are visible in the periphery. Conversely, Fig 1(B) depicts the network structure of the Korean stock market during the global financial crisis in 2008, with the same number of nodes. The network is dense, with only a few detached nodes, and all other nodes are tightly connected, forming a giant cluster with *N*_*_gaint*_ = 290. This suggests a closely coupled network structure, indicating a highly correlated stock market during times of crisis.

### 4.2 N-dependency of network properties

**Fig 2:**
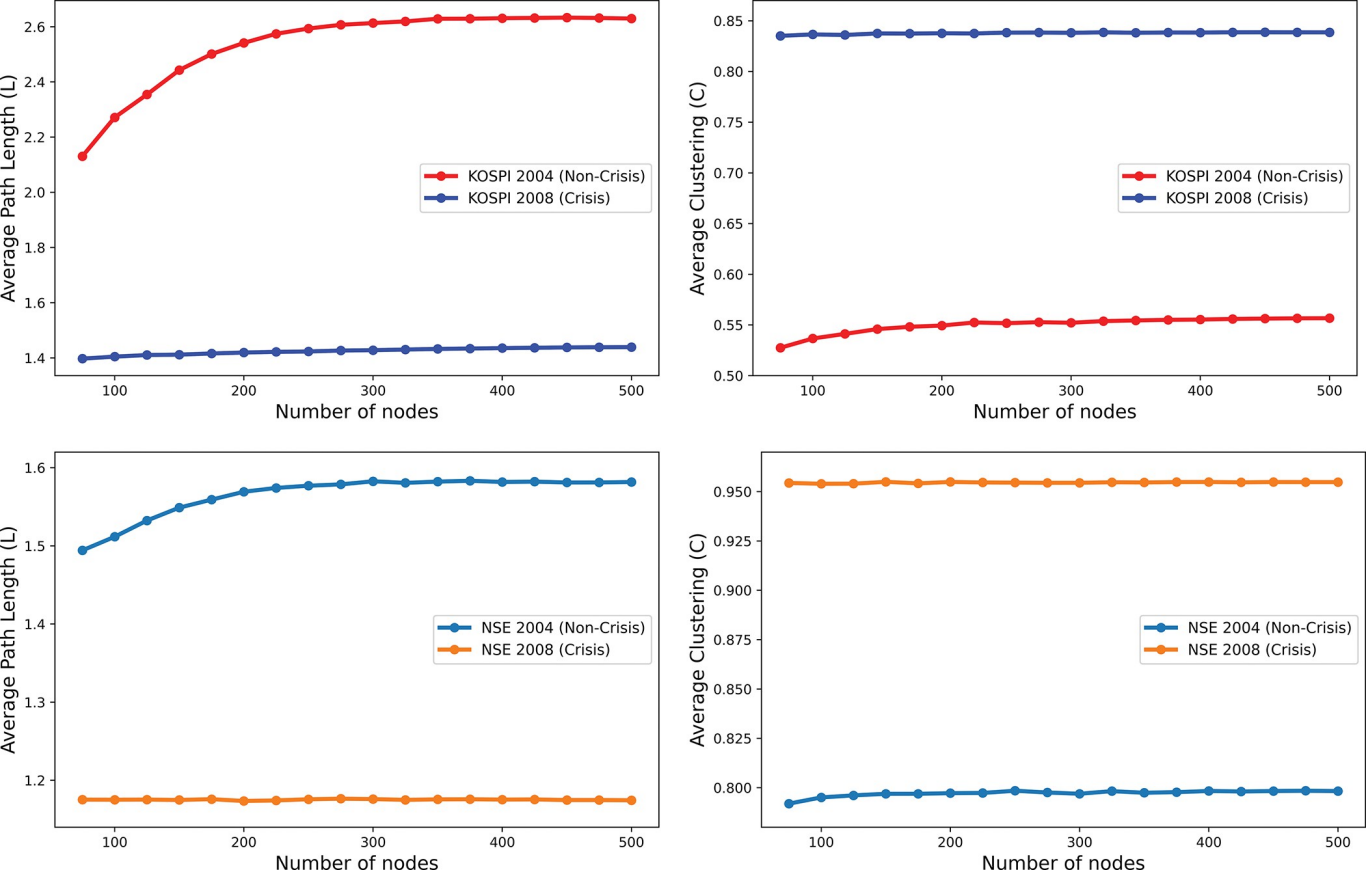
Effects of network size on network measures, specifically, (a) the average shortest path length of KOSPI indices, (b) the average clustering of KOSPI indices, (c) the average shortest path length of NSE indices, and (d) the average clustering of NSE indices. To obtain more precise curves, we averaged these results over 2,000 ensembles.

Fig 2 illustrates the variability of the average shortest path length and the average clustering coefficient as a function of the number of nodes *N* for the years 2004 and 2008. In 2008, South Korea and India experienced the global financial crisis, and to calculate the characteristic quantities of the stock network, we averaged over 2000 configurations. We compared the network properties between the normal period and the crisis period. In Fig 2(A), we observe that for the KOSPI stocks in 2004, the shortest path length L increases significantly with *N*, especially for *N*<200, and it increases slowly for *N*>200. Conversely, the path length *L* for 2008 increases only slightly with the number of nodes, which is barely noticeable in the graph at the given scale. For larger networks, the average path length responds similarly to changes in *N* for both crisis and non-crisis periods.

Furthermore, we observe that the average path lengths of stock networks are significantly smaller during the crisis period than during non-crisis periods, and they are more resistant to changes in *N*. This suggests that networks are more strongly interconnected during the crisis period, and nodes are reachable from each other with fewer steps. We see a similar trend for the average shortest path length of NSE stocks, as depicted in Fig 2(C), but the range of variation is narrower than that of KOSPI stocks. Fig 2(B) illustrates that the average clustering coefficient of KOSPI stocks increases with the number of nodes, as seen in both crisis and non-crisis periods. However, the clustering coefficient for the non-crisis period increases more rapidly than it does for the crisis period, wherein changes in the clustering coefficient are minimal and hardly noticeable in the figure.

The clustering coefficient during the crisis period is higher than that during the non-crisis period, indicating a greater compactness of clusters during the crisis. The average clustering of NSE stocks exhibits similar characteristics to KOSPI stocks, as illustrated in Fig 2(D), but with a narrower range of variation. Conversely, the average shortest path length shows the opposite trend: when the average shortest path length is short, the network is strongly clustered. Finally, by comparing the scales of path length and clustering coefficient in Fig 2(A)–2(D), we observe that the path length is much more sensitive to changes in network size, while the average clustering coefficient of the stock network changes with a smaller margin, making it more robust and less biased by *N*.

### 4.3 Methods for correcting N-dependency of network properties

#### 4.3.1 Normalization by random network

**Fig 3:**
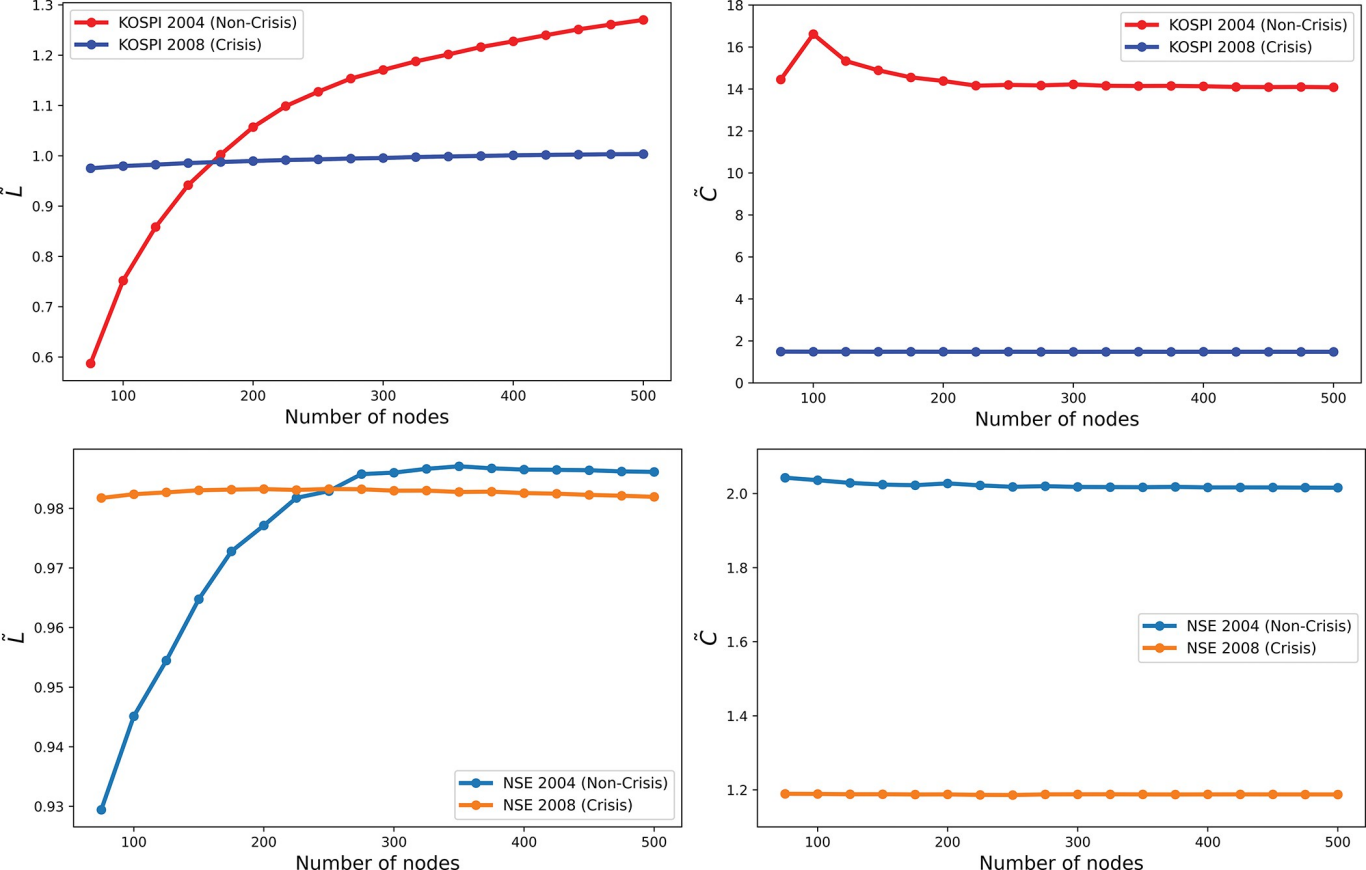
The impact of network size on normalized network measures, based on Erdos-Renyi random graphs, is explored in the following analyses: (a) normalized average shortest path length of KOSPI indices, (b) normalized average clustering of KOSPI indices, (c) normalized average shortest path length of NSE indices, and (d) normalized average clustering of NSE indices. These results were averaged over 2,000 ensembles.

Fig 3 displays the normalized shortest path length (*L*/*L*_*rand*_) and clustering coefficient (*C*/*C*_*rand*_) of KOSPI and NSE stocks compared to Erdos-Renyi random networks. As depicted in Fig 3(A), the normalized shortest path length for KOSPI stocks in 2004 is sensitive to network size, increasing logarithmically with *N*. In contrast, the non-normalized path length varies on a larger scale during the non-crisis period. However, the normalized shortest path lengths in 2008 show slow growth over increasing *N*, reducing the distinction between crisis and non-crisis periods. Similar findings are observed for NSE stocks in Fig 3(C). In Fig 3(B) and 3(D), the normalized clustering coefficients for both KOSPI and NSE stocks in 2004 exhibit greater sensitivity to network size during the non-crisis period compared to non-normalized values. This is demonstrated by the large y-axis scale. However, during the crisis, the normalization process has little impact on clustering coefficients, which change similarly to non-normalized values. The average clustering coefficients throughout the non-crisis period are much higher than those of the crisis period after normalization. Additionally, during the 2008 financial crisis, the normalized path lengths and clustering coefficients of stock networks are close to 1, indicating randomness in stock networks during the crisis.

#### 4.3.2 Normalization by the range of possible values

**Fig 4:**
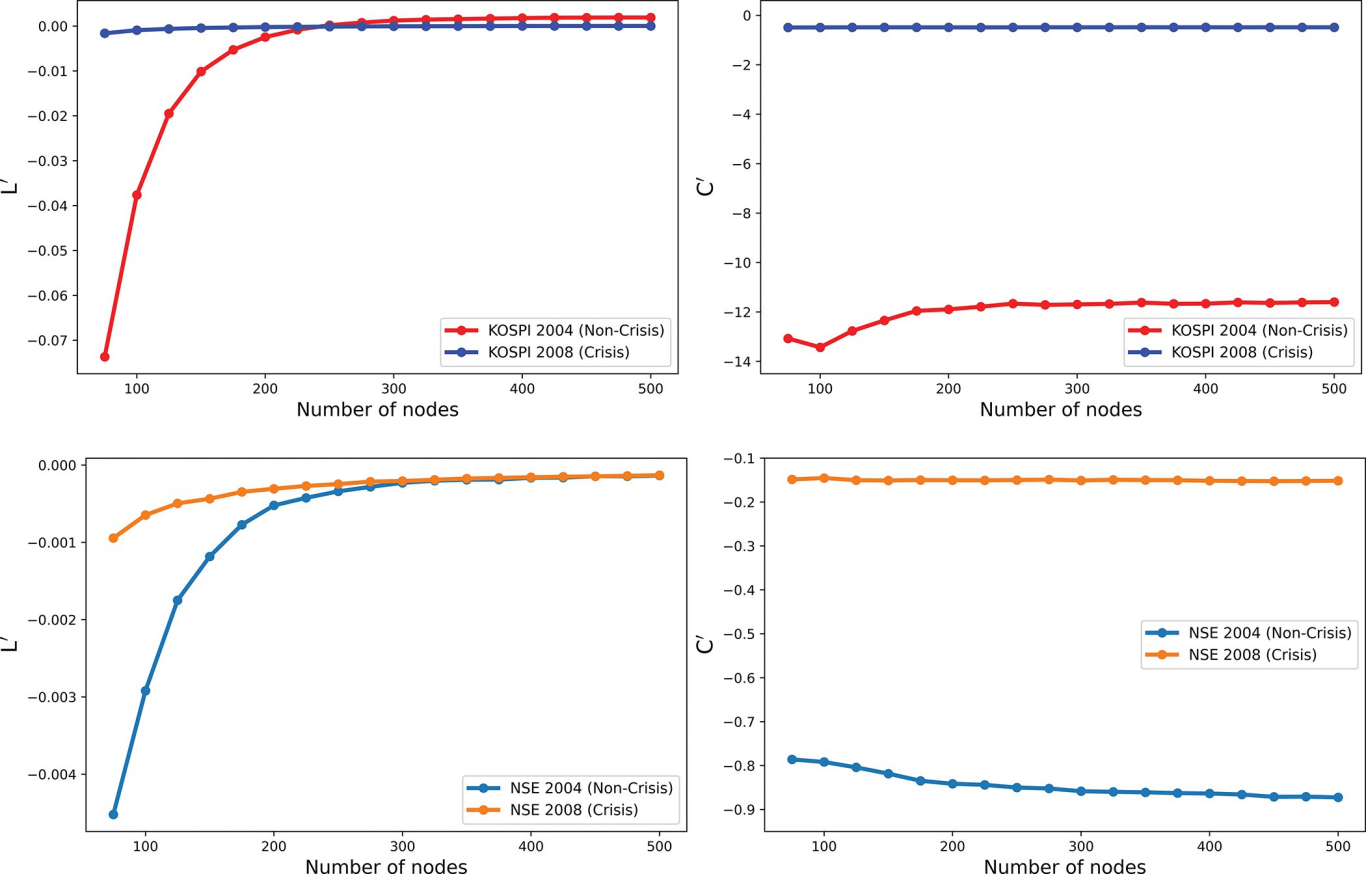
Effects of network size on normalized network measures **(a)** on normalized average shortest path length and **(b)** on normalized average clustering of KOSPI and NSE indices Results are averaged over 2,000 ensembles.

In this technique, the network measures are normalized by the range of possible outcomes between a regular ring lattice network and an Erdos-Renyi random graph, as shown in Eq 6. The result after this normalization is depicted in Fig 4. According to the Fig 4(A) and 4(C), during the non-crisis period, the normalized average path length of KOSPI and NSE stocks grow with *N* when *N* is smaller; however, when *N* is larger, the dependence on *N* is significantly reduced, and the slope of the curve becomes zero when *N* is greater than 200. Also, the dependency of normalized path length is reduced for the crisis period, but this is a slight reduction that is not clearly visible in the figure. But the scale of the y-axis shows that normalizing by the range of possible values reduces the sensitivity of the average path length to *N* substantially in both crisis and non-crisis periods. On the other hand, average clustering remains highly sensitive to changes in the number of nodes, as illustrated in Fig 4(B) and 4(D), despite the range of possible values normalizing it. The normalized clustering coefficient of KOSPI stocks grows with an increasing number of nodes during the non-crisis period, especially between *N* equals 100 and 200, and during the crisis, this dependency remains as before. This trend is almost similar but opposite to the average clustering normalized by the random network in Fig 3(B). The normalized clustering coefficient of NSE stocks decreases with an increasing number of nodes during the non-crisis period, and during the crisis, this dependency remains as before. In summary, normalization by the range of possible values reduces the sensitivity of path length to the variability of *N*, but the crisis period is not distinguishable from non-crisis timelines when N is greater than 200 as path lengths of both periods are almost overlapping after this normalization. However, it makes the clustering coefficient more sensitive to the variability of *N*, particularly in non-crisis situations.

This technique involves normalizing network measures by the range of possible outcomes between a regular ring lattice network and an Erdos-Renyi random graph, as shown in Eq 6. Fig 4 illustrates the results after normalization. During the non-crisis period, the normalized average path length of KOSPI and NSE stocks initially increases with *N* but then plateaus when *N* exceeds 200. Normalizing by the range of possible values substantially reduces the sensitivity of the average path length to *N* in both crisis and non-crisis periods, as indicated by the scale of the y-axis. However, the average clustering remains highly sensitive to changes in the number of nodes, as shown in Fig 4(B) and 4(D), despite the range of possible values normalizing it.

The normalized clustering coefficient of KOSPI stocks increases with an increasing number of nodes during the non-crisis period, particularly between *N* equals 100 and 200, and during the crisis, this dependency remains the same. This trend is almost opposite to the normalized clustering coefficient of the random network in Fig 3(B). The normalized clustering coefficient of NSE stocks, on the other hand, decreases with an increasing number of nodes during the non-crisis period, and during the crisis, this dependency remains unchanged.

In summary, normalizing by the range of possible values reduces the sensitivity of path length to the variability of *N*. However, after normalization, the crisis period is not distinguishable from non-crisis timelines when *N* is greater than 200, as the path lengths of both periods almost overlap. Furthermore, normalizing by the range of possible values makes the clustering coefficient more sensitive to the variability of *N*, particularly in non-crisis situations.

### 4.4 Comparison of the *N*-dependency correction methods

**Fig 5:**
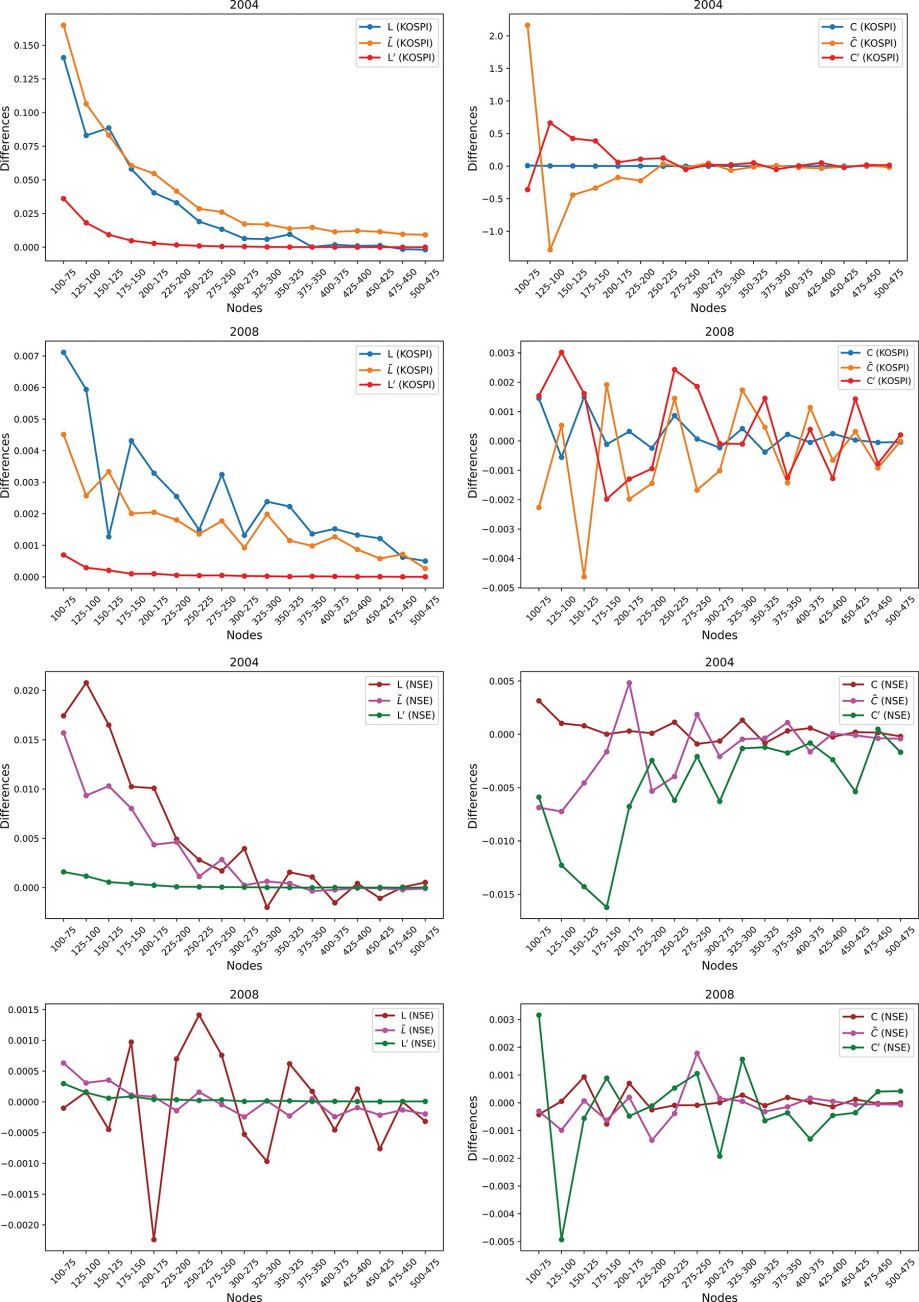
Differences of normalized and non-normalized network metrics for different sized networks during crisis and non-crisis periods. The following are the differences observed: (a) in shortest path length and (b) in average clustering for the KOSPI stocks during the non-crisis period of 2004, (c) in shortest path length and (d) in average clustering for the KOSPI stocks during the crisis period of 2008, (e) in shortest path length and (f) in average clustering for the NSE stocks during the non-crisis period of 2004, and (g) in shortest path length and (h) in average clustering for the NSE stocks during the crisis period of 2008. These differences highlight the sensitivity of network metrics to network size and the impact of normalization on reducing this sensitivity. Additionally, they demonstrate how the financial crisis affected the network metrics differently than the non-crisis period.

Fig 5 presents a comparison of network measures across networks of various sizes, ranging from 75 to 500 nodes in increments of 25. Each point on the graph represents the difference in measures between a network of a given size and the immediately preceding network in terms of size. For instance, the metrics of a network with 75 nodes are subtracted from those of a network with 100 nodes.

During the non-crisis period, Fig 5(A) shows that the differences in average path lengths of KOSPI stocks across various network sizes are significant for smaller networks but become negligible for larger networks. Similar observations are made for $\tilde{L}$, albeit with some residual differences even for larger *N*. Normalizing path lengths by the range of possible values reduces these differences to almost zero, even for small networks. Similar trends are observed during the crisis period, with fluctuating *L* and $\tilde{L}$ values (Fig 5(C)), but little difference between path lengths across networks after normalization. The y-axis scaling in Fig 5(C) reveals that path lengths during the crisis vary within a narrow range.

For NSE stocks, Fig 5(E) and 5(G) depict similar patterns in average path lengths during the non-crisis and crisis periods. The differences in average clustering coefficients for KOSPI stocks across networks are close to zero, regardless of *N* during the non-crisis period, as shown in Fig 5(B). However, normalizations are required for larger *N* values. During the 2008 financial crisis, differences in normalized clustering coefficients for KOSPI stocks fluctuated slightly around zero, while *C* remained close to zero for networks of any size (Fig 5(D)). For NSE stocks, differences in average clustering coefficients fluctuated around zero during both non-crisis and crisis periods but increased significantly after normalization (Fig 5(F) and 5(H)).

Overall, normalizing by the range of possible values significantly reduces the differences in average path lengths between networks of various sizes, making the path length’s sensitivity to *N* negligible. However, there are no significant differences in the average clustering coefficient when no normalization is applied. Moreover, the y-axis scale during the crisis period is much smaller than that during the non-crisis period, indicating less sensitivity of network metrics to *N* during crises.

## 5 Conclusion

We examined how network size affects network measures during both the non-crisis period of 2004 and the global financial crisis of 2008 using the threshold method. Our findings show that during the non-crisis period, the average path length *L* of a network is highly dependent on network size *N*, while less sensitivity to *N* is observed during the crisis. However, the average clustering coefficient remains relatively constant regardless of the size of the network. During a crisis, we found that average path lengths were shorter, and average clustering coefficients were higher than during a non-crisis period, suggesting that network structures become more tightly connected and locally compact during a crisis.

To address the bias introduced by *N* in comparing network measures, we employed various normalization techniques. When the measures of a stock network are normalized with those of a random network, we observed an increase in the sensitivity of both path length and clustering to *N* during the non-crisis period but no noticeable changes during the crisis. Normalizing the path length *L* by the range of possible values reduced its dependence on *N* on a large scale, and there was no bias for *N* greater than 200. However, this normalization technique increased the sensitivity of the clustering coefficient to *N*.

Our study also found that the *N* -dependency of network measures differ during crisis and non-crisis periods. When comparing networks with a large number of nodes, the dependence of both normalized and non-normalized network measurements on *N* is lower. Moreover, during a crisis, the effect of changing *N* on network measures is significantly lower than during the non-crisis period. To compare stock markets of varying sizes with minimal bias, we recommend using normalized average shortest path lengths and non-normalized clustering coefficients when analyzing networks with a large number of nodes.

While our study has made progress in reducing the dependence of network measures on network size, there are still some residual impacts that we were unable to fully eliminate. Our study focused on a sample of up to 500 companies from developing countries over a 1-year time frame using a fixed threshold approach, which has some limitations. We plan to expand our research to include other countries and integrate advanced machine learning and other network construction techniques to deepen our understanding of this fascinating subject. It is essential to consider the effects of *N* -dependent biases in graph measurements when conducting experimental analyses of stock markets.
